# Cerebroplacental Ratio as a Predictive Factor of Emergency Cesarean Sections for Intrapartum Fetal Compromise: A Systematic Review

**DOI:** 10.3390/jcm13061724

**Published:** 2024-03-17

**Authors:** Blanca Novillo-Del Álamo, Alicia Martínez-Varea, Elena Satorres-Pérez, Mar Nieto-Tous, Silvia Bello-Martínez de Velasco, María Victoria García-Florenciano, Carmen Padilla-Prieto, Fernando Modrego-Pardo, José Morales-Roselló

**Affiliations:** 1 Department of Obstetrics and Gynecology, La Fe University and Polytechnic Hospital, Avenida Fernando Abril Martorell 106, 46026 Valencia, Spain; novillo_bla@gva.es (B.N.-D.Á.); satorres_ele@gva.es (E.S.-P.); bello_sil@gva.es (S.B.-M.d.V.); garcia_mariavictoriaflo@gva.es (M.V.G.-F.); padilla_carpri@gva.es (C.P.-P.); modrego_ferpar@gva.es (F.M.-P.); jose.morales@uv.es (J.M.-R.); 2 Department of Pediatrics, Obstetrics and Gynecology, Faculty of Medicine, University of Valencia, 12006 Valencia, Spain; 3 Department of Medicine, CEU Cardenal Herrera University, 12006 Castellón de la Plana, Spain

**Keywords:** cesarean section, delivery, labor, hypoxia, cerebroplacental ratio, intrapartum fetal compromise, Doppler

## Abstract

**Background**: This systematic review aimed to clarify the association between the cerebroplacental ratio (CPR) and emergency cesarean sections (CSs) due to intrapartum fetal compromise (IFC). **Methods**: Datasets of PubMed, ScienceDirect, CENTRAL, Embase, and Google Scholar were searched for studies published up to January 2024 regarding the relationship between the CPR and the rate of CS for IFC, as well as the predictive value of the CPR. **Results**: The search identified 582 articles, of which 16 observational studies were finally included, most of them with a prospective design. A total of 14,823 patients were involved. A low CPR was associated with a higher risk of CS for IFC. The predictive value of the CPR was very different among the studies due to substantial heterogeneity regarding the group of patients included and the time interval from CPR evaluation to delivery. **Conclusions**: A low CPR is associated with a higher risk of CS for IFC, although with a poor predictive value. The CPR could be calculated prior to labor in all patients to stratify the risk of CS due to IFC.

## 1. Introduction

The placenta is the organ in charge of supplying the fetus with enough nutrition to allow its appropriate growth during pregnancy [1], and it also copes with different hypoxic insults [1]. Labor is a physiological but stressful situation for the fetus and the mother [1], characterized by repeated transient episodes of uterine contractions, causing fetal intermittent hypoxia [1]. Each uterine contraction imposes an increase of intrauterine pressure up to 25 to 70 mmHg, resulting in a 25% drop in the partial pressure of oxygen at the fetal side [1]. Multiple adaptive physiological mechanisms are triggered at this stage to cope with this situation. Chemoreflexes are activated in response to fetal hypoxia when utero placental perfusion declines by over 50% [1]. These mechanisms try to centralize the fetal blood flow into the most vital organs of the fetus, such as the brain, the heart, and the adrenals [1]. In this scenario of hypoxic insult, the perinatal and obstetric outcomes result from the balance between placental supply and fetal demands [1]. This balance could be particularly damaged depending on the duration and intensity of labor, as well as the metabolic reserves of the fetus prior to the onset of labor. In case of a mismatch of supply and demand, metabolic reserves diminish, and the fetus suffers hypoxia [1]. As a result, the fetal cardiotocogram becomes abnormal, and fetal well-being cannot be assured [1]. In that situation, an emergency cesarean section (CS) is forced—CS for intrapartum fetal compromise (IFC)—to avoid damage to the newborn [1]. This damage would be mainly neurological damage, as the fetal brain is susceptible to hypoxic injury and oxidative stress or even stillbirth if the hypoxic insult cannot be countered by the adaptive mechanisms [1].

Prior to labor, the functional reserve of the fetus determines the fetal ability to cope with the hypoxia suffered during labor. Consequently, the evaluation of the functional fetal reserve becomes of crucial importance in the prediction of the development of labor and the obstetric and perinatal outcomes. Currently, the usual clinical practice is to evaluate the fetus using the estimated fetal weight (EFW). However, while it is evident that placental function may be decreased in very small fetuses, in cases where growth remains appropriate for gestational age (AGA), which means the fetus weight is above the 10th centile for the gestational age, fetuses may be erroneously classified as “normal” regardless of a similar growth restriction due to a later placental dysfunction. This dysfunction has not yet impacted the fetus’s weight because of a later onset. As these fetuses, genetically determined to achieve a higher weight percentile than the one they have, do not reach their growth potential [2], the obstetric outcome may be unexpectedly poor and may have similar adverse consequences.

Concerning physiological adaptation to stress and hypoxemia, cerebral redistribution is one of the first mechanisms of compensation [1]. This could be detected by evaluating the middle cerebral artery (MCA) pulsatility index (IP) Doppler and its ratio to the umbilical artery (UA) IP Doppler: the cerebroplacental ratio (CPR). The CPR could behave as a marker of failure to reach the growth potential, regardless of fetal size. The CPR could be a way of evaluating the fetus using fetal hemodynamics, as it could reflect the fetal cerebral redistribution in response to hypoxemia, as this may occur at all centiles of fetal weight [2]. To correctly calculate the fetal Doppler, convex probes of 2–8 MHz should be used during fetal quiescence, including voluntary maternal suspended breathing, in the absence of fetal tachycardia, and keeping the insonation angle with the examined vessels as close to zero as possible [3,4]. The UA IP should be determined from a free-floating cord loop. The MCA IP should be obtained in a transversal view of the fetal head at the level of its origin from the circle of Willis. The ICP is calculated as the ratio of the MCA IP to the UA IP Doppler index [4].

Systematic reviews have been published evaluating the predictive capacity of the CPR for adverse perinatal outcomes [5,6]. Nonetheless, none of them have considered the relationship between the CPR and emergency CS for IFC as a single outcome—an issue that could be useful in daily obstetric practice. Thus, the authors aimed to shed light on the ongoing controversy of the relationship between the CPR and CS for IFC, as well as the predictive value of the CPR. Additionally, the authors ought to determine if the CPR could be used in daily clinical practice to predict emergency CS for IFC and when it should be calculated. A systematic review of the literature regarding the CPR and CS for IFC was designed for that purpose.

## 2. Material and Methods

This systematic review was carried out according to PRISMA guidelines [7].

### 2.1. Literature Search

Five reviewers searched PubMed, ScienceDirect, CENTRAL, Embase, and Google Scholar databases, looking for the literature for this systematic review. The keywords used were “cerebroplacental ratio”, “Doppler”, “intrapartum fetal compromise”, “emergency cesarean section”, and “placental dysfunction”.

ZOTERO (v. 6.0.30) was the bibliographic manager used to arrange the articles and eliminate the duplicates. The literature search was performed, and all studies published up to 1 January 2024 were initially selected.

### 2.2. Eligibility Criteria

The inclusion criteria were the following: randomized controlled trials, cohort studies, longitudinal studies, case-control studies, ecological studies, or cross-sectional studies regarding the relation between the CPR and the primary outcome: CS for IFC. IFC was defined as the presence of an abnormal intrapartum fetal heart rate or intrapartum fetal scalp pH below 7.20. There were no secondary outcomes.

The exclusion criteria were the following: review articles, studies including multiple gestations (twin pregnancies or more), elective CS (for any reason), studies with less than 50 women included in the sample, or studies not including CS as an independent outcome (composed fetal or perinatal outcomes were excluded). Studies for which the original full manuscript could not be found were also excluded. There were no language or geographical area restrictions. Two more researchers double-checked that the selected abstracts met the criteria. Disagreements were resolved by discussion and consensus. Data were then collected by reading the full articles that were finally included.

### 2.3. Data Extraction

The following data were extracted from all the studies selected for the systematic review: first and last author details, article publication year, journal where it was published, time of developing the inclusion of the patients in the study, country of location of the study, sample size (N), study design and type of statistical analysis used for presenting the results, inclusion criteria, outcomes, gestational age at CPR examination, time interval from calculating the CPR to delivery, methods for CPR examination, and results and conclusion of the authors.

### 2.4. Assessing the Quality of the Studies and the Risk of Bias

The authors used the Newcastle Ottawa scale [8] to assess study quality. The Newcastle Ottawa scale has three domains: selection of study population, comparability, and outcomes [8]. Every study is evaluated in each domain, and they are given stars depending on the adequacy of the task in the different domains. In the selection category, the authors principally evaluated the representativeness of the cohort, exposed or not. Regarding the comparability category, the authors assessed if the cohorts used were comparable based on the design or analysis. In the outcome domain, the authors evaluated the assessment of the outcome and the adequacy of the follow-up of the cohorts in terms of the outcome occurring, if that happened [8].

Study quality was examined independently by four authors. To avoid bias, four authors established a consensus for assessing the risk of bias in individual studies at both the study and outcome levels. Disagreements were solved by consensus. The biases detected are described in the limitations portion of the Discussion section.

## 3. Results

**Figure 1:**
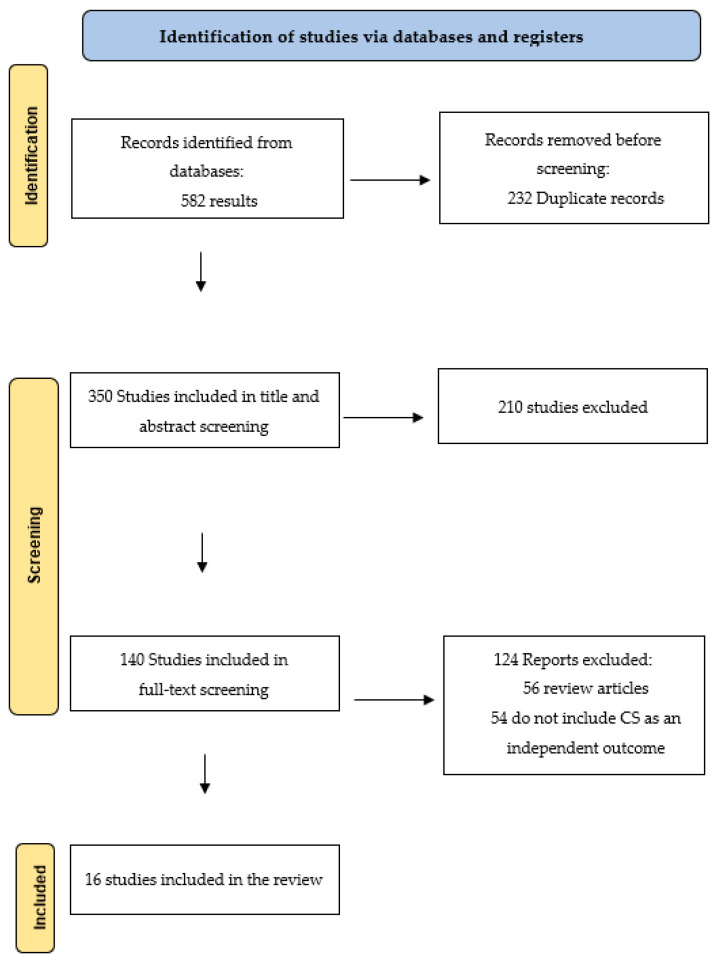
PRISMA: Preferred Reporting Items for Systematic Reviews.

The search, performed according to the material and methods section, identified 582 articles in the databases. A total of 16 studies that fulfilled the inclusion criteria were ultimately included in this systematic review. All the studies together involved 14,823 patients. The PRISMA flow chart shows how the articles were selected (Figure 1).

All the studies included were observational (Table 1). Eleven of them were prospective, and five were retrospective designs (Table 1). Two of the studies included were multicenter [3,9]. The way of onset of labor was not taken into account as an inclusion criterion. Some of the studies included only elective inductions of labor [10,11,12,13,14,15], while others included only spontaneous onset of labor [9], both elective and spontaneous, or did not specify the means of onset of labor [2,3,16,17,18,19,20,21] (Table 1). Two of the studies excluded patients with previous uterine scars in their examination of previous CSs, including those resulting from myomectomies [9,11] (Table 1).

Given that the outcome of this review was emergency CS due to IFC, the studies that included other variables in the composite outcome were excluded [22]. In all selected studies, the CPR was independently related to the risk of CS due to IFC. The overall results are shown in Table 1.

Most authors concluded that low CPR values were associated with a higher risk of CS for IFC [9,12,13,15,17,20]. However, the statistical analysis was heterogeneous concerning the way of presenting results in the different studies included in the systematic review. Some authors used odds ratios (ORs) to express the higher possibility of CS for IFC due to a low CPR: OR 2.6 [13], OR 2.57 [19], and OR 10.3 [4], while other authors indicated the ability of the CPR to predict CS for IFC. In these last cases, the results were also heterogeneously expressed. Some authors used the area under the curve (AUC): AUC 0.73 [16], AUC 0.71 [3], and AUC 0.82 [2], while other authors suggested the existence of a poor prediction, showing the sensitivity (26%), specificity (87%), positive likelihood ratio (2.0), and negative likelihood ratio (0.85) of the measurement of the CPR to predict CS for IFC [10].

Substantial heterogeneity was also found regarding the population studied in the different articles included (Table 1). Some studies included only low-risk pregnancies [9,10,11,14,20,21], while others studied specifically small gestational-age fetuses [4,18,19]. Other studies did not select the population and included a heterogeneous but representative sample of patients of the entire population with all the different EFWs and placental functions [2,3,17].

The heterogeneity previously described regarding the statistical analysis and the population included in the different studies of this systematic review precluded the possibility of performing a meta-analysis.

Three studies from Morales-Roselló et al. fulfilled the inclusion criteria for this systematic review [2,3,16]. All of them demonstrated an association between a low CPR Doppler and a higher rate of CS for IFC. Nonetheless, in one of those studies, the authors placed the MCA PI multiples of median (MoM) as the best predictor of adverse perinatal outcome (AUC = 0.76) versus CPR MoM (AUC = 0.73) [16].

Several authors have tried to enhance the ability of the CPR to predict CS due to IFC by including other variables in different multivariate models, like gestational age, amniotic fluid index [15], parity [18], Bishop score [19], and EFW [11]. However, no combination obtained a perfect prediction [9,12,13,15,17,20]. In a prospective cohort of 384 patients, Lu et al. concluded that the combination of the CPR, nulliparity, and EFW did not show any advantage [11]. Nevertheless, these variables behaved as independent predictors of CS for IFC [11]. In another study, with a multicenter cohort of 5193 patients, the authors improved the prediction by adding EFW and type of labor onset (AUC 0.71, 0.73, and 0.75, respectively) [3]. However, the utility of the EFW differs according to the different studies. Some studies concluded that the EFW is a crucial variable for the prediction of CS for IFC [12], while others concluded that EFW is less critical than the CPR [16].

Makles and Wilczyński, agreeing with the relationship between low CPR Doppler values and the higher rate of CS for IFC, concluded that CPR calculation might reduce the number of “hastily” conducted CSs [15].

Regarding the interval between CPR examination and delivery, a multicenter study with a total of 5193 patients studied the accuracy of the prediction with different time intervals to delivery: 0–1, 2–7, 8–14, and 15–30 days. The authors concluded that the best prediction was achieved when the CPR was evaluated prior to labor—precisely when it was calculated 0 to 1 day before labor (AUC 0.71). A trend of lower accuracy with higher intervals of examination from CPR to delivery can be observed [3]. Nevertheless, this review included studies with different intervals from examination to delivery (Table 1): one week [10], two weeks [13], or even four weeks [18].

## 4. Discussion

After systematically reviewing the literature on the relationship between a low CPR and the rate of CS for IFC, the authors of this systematic review found that fetuses with low functional reserves at the beginning of labor are at risk of CS for IFC [10]. The CPR Doppler is probably the best marker for the interrogation of such reserves, as low CPR values at the end of pregnancy, although with a poor predictive value [9,10,13,17], have been associated with a higher risk of CS for IFC [9,12,13,17,20] at delivery. The CPR represents the ability to diagnose placental dysfunction before any significant decrease in fetal size occurs [21]. Therefore, fetuses that begin labor with a low CPR, regardless of their estimated size, are likely to be at higher risk of CS for IFC [9,12,13,17].

Regarding pregnancy control, many obstetricians are confident that a normal fetus weight and a normal UA IP, especially in AGA fetuses, warrant fetal well-being [2]. However, since there are cases with normal UA IP and low CPR, the MCA IP should also be evaluated in order to allow the calculation of the CPR [4,19]. Calculating just the AU IP Doppler may give the obstetrician false reassurance.

Additionally, the existence of small-for-gestational-age fetuses with a pathological CPR despite a normal UA IP is associated with the highest risk of CS delivery [4]. Therefore, among fetuses diagnosed as small for gestational age, there is a subgroup with true growth restriction and mild placental insufficiency that has begun to centralize its blood flow, represented by pathological ICP, but with a normal UA IP Doppler [4].

Therefore, fetuses with low CPR values, regardless of the estimated fetal size, should be considered late-onset growth restriction fetuses and should have their own induction protocols [2]. On the other hand, fetuses considered small for gestational age but with a Doppler within normality and growth expectations according to their percentiles from the beginning of pregnancy should be considered constitutionally small, growing according to their genetic potential. They should not follow the same protocols as real growth restriction fetuses associated with placental dysfunction [2].

Our results also suggest that it is essential to consider the time interval between examination and delivery, as the CPR’s prediction ability worsened with the lengthening of this interval [3]. In this regard, the different intervals from examination to delivery of the studies included in the review (Table 1) might be a reason for the different CPR predictive values observed [9,10,13,17]. However, another explanation might be the heterogeneity of the included patients (Table 1). The heterogeneity in both features—the moment when the CPR is calculated related to the labor and the group of patients for whom the CPR is calculated—holds the key to the different numerical predictive values presented by the studies included in the systematic review. This is the reason that has precluded the authors from accomplishing a meta-analysis.

Nonetheless, the obstetric result is a combination of fetal metabolic reserves, represented by the CPR, and the intensity of the hypoxia during labor, represented by the combination of multiple variables: the onset and length of labor, the different cervical conditions of the mother, and the variable myometrium response of the uterus to oxytocin [14]. Unfavorable cervical conditions, described as a low Bishop score; induced labor; and the inadequate response of the uterus to oxytocin, lead to more prolonged labor. Therefore, the fetus will suffer from hypoxia for a more extended period of time. Furthermore, an excessive response to oxytocin of the uterus without leading the fetus to oxygenate between contractions would lead to acute intense hypoxia in the fetus. In both situations, the fetus would need better physiological reserves to cope [2]. Thus, the combination of unfavorable maternal soft tissues, induced labors, and low fetal physiological reserves would result in the highest risk of fetal hypoxia during labor (due to intensity, length of the labor, or both) and, therefore, a higher risk of emergency CS due to IFC [14].

Concerning the clinical application of the CPR, this remains unclear, as the CPR’s association with a higher frequency of CS for IFC [9,12,13,17,20] might also depend, as the authors discussed before, on other parameters, like the onset and length of the labor, the different cervical conditions of the mother, and the variable myometrium response of the uterus to oxytocin [14]. Nowadays, the clinical guidelines of obstetric scientific societies do not include induction for a low CPR without other fetal alterations. Nonetheless, according to these data, its measurement might be used to stratify the risk of CS due to IFC prior to labor.

Prior systematic reviews have sought to ascertain the accuracy of the CPR in predicting adverse perinatal and neurodevelopmental outcomes [5,6]. Still, none of them have assessed the relationship between the CPR and emergency CS due to IFC as a single outcome. One of the reviews used a very select population with only fetuses with suspected growth restriction [6]. After including 22 studies involving 4301 women, the authors concluded that the CPR appears to be useful in predicting perinatal death in pregnancies with suspected growth restriction with an ROC curve of 0.83 [6]. Other systematic reviews, including 128 studies that involve 47,748 women with pregnancies at any type of risk, ought to assess the predictive accuracies of the CPR and MCA IP Doppler in predicting adverse perinatal outcomes, as well as if the calculation of the CPR Doppler adds any value to the calculation of the UA IP Doppler [5]. The authors concluded that the calculation of the CPR Doppler could add value to UA IP Doppler assessment in terms of predicting adverse perinatal outcomes [5]. However, the authors acknowledged that it remains unclear to which subgroup of pregnant women the calculation should be applied (Field [5]. Both systematic reviews concluded that randomized controlled trials are needed to assess the importance of the CPR before incorporating its measurement in clinical decisions [5,6].

The strength of this systematic review is its novelty, as this is the first systematic review studying the relationship between the CPR and emergency CS due to IFC as a single outcome. The limitations are characterized by the heterogeneity of the studies included, regarding different time intervals from examination to delivery, diverse populations, and distinct methodologies. Furthermore, another limitation would be the presence of possible underreported studies. The vast majority of studies published are performed in First World areas. In deprived areas, where the birth rate is higher, Doppler calculations are not usually performed due to the lack of technical resources or overworked staff. This would be a limitation for generalizing future protocols, including Doppler studies.

## 5. Conclusions

To conclude, additional studies are needed to clarify the association between the CPR and risk of CS for IFC and to determine whether evaluating the CPR before labor results in clinical benefits.

## Figures and Tables

**Table 1:** Studies included in the systematic review.

First Author, Year of Publication	Time	Country	Design of the Study	Number of Patients (N)	Inclusion Criteria	Interval Time from CPR Measurement to Delivery	Results—Conclusion Regarding CST for IFC
Ortiz, 2023 [10]	03/2012 to 12/2017	Germany	Retrospective observational study	314	41 + 0 to 41 + 6 weeks of pregnancy elective induction of labor AGA	Within one week	The predictive value of CPR was poor (sensitivity = 26%, specificity = 87%, positive LR = 2.0 and negative LR = 0.85).
Morales- Roselló, 2022 [16]	Does not say	Spain	Prospective observational study	182	34 to 41 weeks of pregnancy	Within 24 h	MCA PI was the best predictor (AUC = 0.76), compared to CPR (AUC = 0.73) and VPR (AUC = 0.71).
Morales- Roselló, 2022 [3]	Does not say	Spain UK Italy	Multicenter retrospective observational study	5193	35 to 41 weeks of pregnancy	Within one month	The predictive ability of CPR worsened with the interval to delivery. The best prediction was obtained prior to labor and by adding information related to EFW and type of labor onset (AUC = 0.75).
Morales- Roselló, 2021 [2]	Does not say	Spain	Retrospective observational case-control study	254	32 to 41 weeks of pregnancy	Within 24 h	CPR was a moderate predictor (AUC = 0.82). The predictive abilities of a multivariable model did not differ from the CPR alone.
Lu, 2021 [11]	01/2020 to 12/2020	USA	Prospective observational study	384	>37 weeks of pregnancy Inductions of labor without uterine scars	“Before induction of labor”	Nulliparity, small for gestational age and CPR < 10th centile are independent predictors. However, the addition of CPR could not significantly improve the screening accuracy.
Günay, 2021 [12]	12/2018 to 10/2019	Istanbul	Prospective observational study	145	≥37 weeks of pregnancy Inductions of labor Bishop scores of 5 or less	Within 8 h	Abnormal CPR associates with CS in scheduled induction of labor; as well as normal CPR, with later labor induction and higher UA pH.
Ho, 2020 [13]	01/2000 to 04/2017	Australia	Retrospective observational study	2920	≥37 weeks of pregnancy Inductions of labor	Within 2 weeks	At term, the CPR is an independent risk factor for CS IFC regardless of fetal weight. However, fetal size is a more important variable.
Fiolna, 2019 [17]	05/2016 to 07/2018	UK	Prospective observational study	1902	≥37 weeks of pregnancy	Within 24 h	Low CPR is associated with increased risk of CS for IFC, but the addition of CPR did not improve the performance of screening.
Dall’ asta, 2019 [9]	01/2016 to 07/2017	Italy Spain	Multicenter prospective observational study	562	≥37 weeks of pregnancy Spontaneous onset of labor without uterine scar	At admission	Low CPR is associated with a higher risk of IFC; however, it is a poor predictor of adverse perinatal outcome.
Fratelli, 2018 [14]	Does not say	Italy	Prospective observational study	151	Low risk pregnancies Inductions of labor AGA	Within 3 h	CPR itself is unlikely to predict IFC as there are other intrapartum factors influencing (cervical conditions, uterine response to induction, length of labor, etc.).
Makles, 2017 [15]	Does not say	Poland	Retrospective observational study	130	41 to 42 weeks of pregnancy Inductions of labor	Doesn’t say	Calculating CPR and others might allow to avoid making early decisions on performing labor induction and reduce the number of hastily conducted CS.
Kalafat, 2018 [18]	1999 to 2015	UK	Prospective observational study	927	≥37 weeks of pregnancy SGA	Within 4 weeks	CPR (OR = 0.38) and others are independently related with the risk of operative delivery for IFC.
Garcia-Simon, 2015 [19]	03/2007 to 11/2013	Spain	Prospective observational study	164	Late- onset SGA normal UA I PI Doppler	Within 24 h	Bishop score and CPR improves the ability to predict overall CS, emergency CS for IFC and neonatal admission.
Prior, 2015 [21]	03/2011 to 03/2014	UK	Prospective observational study	775	≥37 weeks of pregnancy low-risk pregnancies AGA excluding placental disfunction	Prior to active labor	CPR < 0.6765 MoM were at increased risk of IFC. Low negative predictive value was observed for fetal compromise.
Prior, 2013 [20]	Does not say	UK	Prospective observational study	400	≥37 weeks of pregnancy low-risk pregnancies AGA excluding placental disfunction	Immediately prior to active labor	CPR can identify fetuses at high and low risk of IFC, and may be used to risk stratify pregnancies before labor.
Cruz- Martínez, 2011 [4]	01/2008 to 05/2010	Spain	Prospective observational study	420	210 term SGA with normal UA PI and 210 control participants matched by gestational age	Within 24 h	Evaluation of brain Doppler indices before labor induction discriminates SGA fetuses at high risk of CS for IFC and neonatal acidosis.

CS: cesarean; CPR: cerebroplacental ratio; VPR: vertebroplacental ratio; IFC: intrapartum fetal compromise; AGA: adequate weight for gestational age; SGA: small for gestational age; UA: umbilical artery; MCA: middle cerebral artery; PI: pulsatility index; OR: odds ratio; LR: likelihood ratio.

## Data Availability

Not applicable.

## Abbreviations

cesarean section (CS); intrapartum fetal compromise (IFC); estimated fetal weight (EFW); appropriate for gestational age (AGA); pulsatility index (IP); middle cerebral artery (MCA); umbilical artery (UA); odds ratio (OR); area under de curve (AUC); multiples of the median (MoM).
